# Adult-sourced fecal microbiota transplantation ameliorates early-life antibiotic-induced intestinal immune stress in juvenile mice associated with the modulation of the TLR4/MyD88 signaling pathway

**DOI:** 10.1371/journal.pone.0359634

**Published:** 2026-10-13

**Authors:** Xiang Qian, Yanjing Shen, Xianzhong Luo, Siqin Zhang, Li Wang, Hualin He, Bin Wang

**Affiliations:** 1 Department of Clinical Laboratory, Hangzhou Traditional Chinese Medicine Hospital Affiliated to Zhejiang Chinese Medical University, Hangzhou, China; 2 Department of Pediatrics, Hangzhou Traditional Chinese Medicine Hospital Affiliated to Zhejiang Chinese Medical University, Hangzhou, China; Universidad Nacional Autonoma de Mexico Instituto de Biotecnologia, MEXICO

## Abstract

Early-life antibiotic (ABX) exposure profoundly disrupts gut microbiota assembly, triggering long-lasting intestinal immune stress. While fecal microbiota transplantation (FMT) is a promising intervention, its immunological and ecological mechanisms in the juvenile gut remain underexplored. In this study, juvenile mice were subjected to a broad-spectrum ABX cocktail for 8 days followed by 4 days of adult-sourced FMT. 16S rRNA sequencing (n = 6/group) revealed that ABX drastically altered the microbial landscape, causing a severe relative reduction of core commensals and an opportunistic expansion of Bacteroides. Subsequent molecular analyses on day 13 (n = 3/group) demonstrated that this dysbiosis significantly upregulated the mucosal TLR4/MyD88 signaling axis and effector cytokines IL-17 and IL-22. Strikingly, adult FMT administration effectively attenuated the TLR4 cascade and reduced pro-inflammatory cytokine transcript levels while immunoregulatory IL-10 transcripts remained partially elevated. Ecologically, FMT did not merely revert the gut to its naive juvenile baseline. Instead, it suppressed Bacteroides overgrowth and established a distinctly new, highly complex community enriched with putative mature, adult-signature taxa such as *Parabacteroides*, *Tannerellaceae*, and *Desulfobacterota*. Interestingly, the downregulation of TLR4 occurred concurrently with the engraftment of these Gram-negative, LPS-bearing taxa. Collectively, adult-sourced FMT ameliorates ABX-induced immune stress not by restoring the original microbiota, but by driving the juvenile gut towards a putatively mature, structurally distinct, and potentially immunologically tolerant ecological state.

## Introduction

The neonatal and juvenile periods represent a “critical window” for the assembly of the gut microbiota, a process that occurs in parallel with the maturation of the host immune system. This early-life microbial succession is essential for the establishment of mucosal homeostasis and the programming of systemic immune tolerance [1]. However, this delicate process is highly susceptible to external perturbations, most notably broad-spectrum antibiotic (ABX) exposure [2]. Clinical and experimental evidence suggests that ABX-induced dysbiosis in early life is linked to long-term immunological consequences, including an increased risk of asthma, obesity, and inflammatory bowel diseases later in life [3–5].

Research indicates that antibiotics such as streptomycin and ampicillin significantly alter gut microbiota composition, and their combined use substantially reduces the abundance of *Akkermansia muciniphila* — a bacterium crucial for maintaining gut health, and increases the presence of harmful bacterial genera like *Citrobacter*, thereby exacerbating intestinal inflammation and diarrhea symptoms [6]. Antibiotic-associated diarrhea (AAD), a common adverse reaction to antibiotic therapy, is typically caused by a profound collapse of intestinal flora diversity. For instance, the combination of gentamicin and cefradine significantly increases the abundance of Proteobacteria and Actinobacteria in the gut while reducing the abundance of *Lactobacillus* [7]. This change not only weakens the gut's colonization resistance but also damages the intestinal mucosal barrier function, thereby exacerbating AAD symptoms. While many studies focus on pathogen overgrowth [8], the broader ecological collapse and subsequent “immune stress” remain understudied in the pediatric context.

To mitigate the effects of AAD, the use of probiotics is considered a potentially effective strategy. Studies have demonstrated that specific probiotic strains, such as *Lactobacillus rhamnosus GG* and *Saccharomyces boulardii*, can prevent and treat AAD by restoring the balance of the gut microbiota [9,10]. However, the use of probiotics also poses safety concerns, particularly in immunocompromised patients, where it may increase the risk of severe infections [11]. Despite the regenerative capacity of the gut, antibiotic-disrupted microbiota often fail to recover their original diversity, leading to a state of persistent immune “stress.” Fecal Microbiota Transplantation (FMT) has emerged as a robust strategy for ameliorating microbial ecology. While age-matched FMT is often used, the use of adult-sourced donor microbiota provides a unique “mature template” [12,13]. Adult microbiota are generally more stable and possess a more comprehensive functional gene repertoire, which may provide a competitive advantage for colonization and a more robust “educational signal” for the developing juvenile immune system [14].

Studies have demonstrated that antibiotic exposure induces structural and functional changes in the gut, including loss of intestinal neurons and increased intestinal permeability [15]. These changes may exert long-term effects on overall gut health, particularly in children whose immune systems are not yet fully developed. The Toll-like receptor 4 (TLR4)/ Myeloid differentiation primary response 88 (MyD88) signaling pathway serves as a primary interface between gut microbes and innate immunity [16,17], its overactivation by microbial-derived patterns like lipopolysaccharides (LPS) is a hallmark of intestinal immune stress [18]. These specific cytokines (IL-17, IL-22, and IL-10) were evaluated because IL-17 and IL-22 are primary effectors of mucosal antibacterial immunity and inflammation, whereas IL-10 is critical for maintaining immune tolerance [19–21]. In our pilot experiments, we observed a marked up-regulation of the TLR4/MyD88 pathway in the murine gut following antibiotic administration, which served as the rationale for the current study.

In this study, we investigated whether adult-sourced FMT could alleviate early-life ABX-induced immune perturbation associated with the modulation of the TLR4/MyD88 axis, providing a potential therapeutic framework for pediatric recovery.

## Materials and methods

### Animals and experimental design

Specific pathogen-free (SPF) juvenile C57BL/6 mice (2–3 weeks old, male) and adult C57BL/6J mice (8 weeks old, male) were obtained from Shanghai Slake Experimental Animal Co., Ltd. All mice were housed in a temperature-controlled environment (22 ± 2°C) with a 12-hour light/dark cycle and provided with free access to sterile water and food. All animal experiments were approved by the Institutional Animal Care and Use Committee of Hangzhou Hunter Testing Biotechnology Co., Ltd (ethical approval number: IACUC/HTYJ-11746–33).

To ensure animal welfare and minimize suffering, humane endpoints were strictly established and monitored. Animal health, behavior, and body weight were monitored twice daily by trained research staff. The specific criteria for humane euthanasia included severe lethargy, inability to eat or drink, ruffled fur, abnormal posture, or rapid weight loss exceeding 20% of the baseline body weight. If any animal reached these criteria, it was to be euthanized immediately (within 1 hour) via CO2 asphyxiation followed by cervical dislocation. Throughout the 12-day experimental period, all efforts were made to minimize distress, including the provision of easily accessible food and specialized housing. A total of 18 juvenile mice were used in this study. No animals died or required early euthanasia prior to the planned experimental endpoint. At the conclusion of the experiment, all remaining animals were humanely euthanized.

After one week of acclimatization, 18 juvenile male C57BL/6 mice were randomly assigned to three groups (n = 6 per group):

- Control Group (CON): Received no treatment.
- **Model Group (EXP):** Mice were administered a broad-spectrum antibiotic cocktail via oral gavage twice daily for 8 consecutive days to induce gut dysbiosis. The cocktail consisted of Ampicillin (100 mg/kg; S17018, USP grade), Gentamicin (50 mg/kg; S17024, USP grade), and Vancomycin (50 mg/kg; V30292, USP grade). Following antibiotic treatment, mice received sterile PBS via gavage for 4 days.
- **FMT Group (FMT):** Mice received the same 8-day antibiotic treatment as the EXP group, followed by oral gavage of fecal microbiota suspension (0.1 mL/mouse) once daily for 4 days.

### Preparation of fecal microbiota suspension

Donor feces were collected from healthy, untreated 8-week-old adult C57BL/6J mice. To minimize inter-individual variation, feces were pooled from 5 healthy adult C57BL/6J mice housed in the same batch. Under sterile conditions, fresh fecal pellets were collected and immediately suspended in chilled sterile PBS. The suspension was homogenized and centrifuged at low speed to remove large particulate matter. The supernatant was collected, and the bacterial concentration was adjusted to an optical density (OD_600_) of approximately 0.5, corresponding to roughly 10^9^ CFU/mL. The prepared FMT suspension was used for gavage within 2 hours.

### 16S rRNA gene sequencing and bioinformatics analysis

Fresh fecal samples were collected at the end of the experiment and stored at −80°C. Total genomic DNA was extracted, and the V3-V4 hypervariable regions of the 16S rDNA gene were amplified. Paired-end sequencing (2⊆250 bp) was performed on the Illumina platform with automated cluster generation and dual indexing, following the manufacturer’s protocols. All raw sequencing data generated in this study have been deposited into the NCBI BioProject database with the accession number PRJNA1447757.

### Data preprocessing and OTU clustering

Paired-end sequencing was performed on the Illumina platform. Raw data were processed to merge forward and reverse reads. Sequences containing ambiguous “N” bases were filtered, and only those with a length greater than 200 bp were retained. Following quality filtering and the removal of chimeric sequences, high-quality sequences were clustered into Operational Taxonomic Units (OTUs) at a 97% identity threshold using VSEARCH (v1.9.6). Taxonomic assignment was performed using the RDP classifier (Naive Bayes algorithm) against the SILVA database (v138). Community composition statistics were then calculated at various taxonomic levels.

### Diversity and community structure analysis

To account for differences in sequencing depth, the OTU table was rarefied based on random sampling. Alpha diversity indices (Shannon and Chao1), along with Rarefaction curves and Rank-Abundance graphs, were generated to evaluate species richness and evenness. Beta diversity was assessed and visualized using Principal Component Analysis (PCA, based on OTU abundance), Principal Coordinate Analysis (PCoA), and Non-metric Multidimensional Scaling (NMDS) based on Bray-Curtis and (un)weighted UniFrac distances. Furthermore, a UPGMA (Unweighted Pair Group Method with Arithmetic Mean) clustering tree was constructed to compare similarities between samples.

### Statistical analysis

Significant differences in microbial community structure between groups were evaluated using Permutational Multivariate Analysis of Variance (PERMANOVA/Adonis) via the ‘vegan’ package. The Wilcoxon rank-sum tests was used to compare univariate data, such as alpha diversity indices and the relative abundance of specific taxa. LEfSe (Linear discriminant analysis Effect Size) was employed to identify differentially abundant taxonomic biomarkers across groups, with the logarithmic LDA score threshold set to > 3.0 and a significance level of *p* < 0.05. Furthermore, a LEfSe cladogram was generated to visualize the phylogenetic distribution of these structural biomarkers, restricting the taxonomic representation from the phylum down to the genus level. All statistical analyses and visualizations were performed using R (v4.4.1) with packages including ‘ggplot2’ and ‘vegan’.

### Tissue harvesting and preparation

Mouse intestinal tissues were harvested on day 13 following humane euthanasia. The digestive tract was traced to locate the cecum. An ileal segment upstream and a colon segment downstream of the cecum were excised, and surrounding mesenteric tissues were carefully removed. The intestinal segments were opened longitudinally, washed 2–3 times in phosphate-buffered saline (PBS), and blotted dry on filter paper. The collected distal colon tissues (n = 3 distinct mice per group, selected randomly from each cohort to provide preliminary molecular data while conserving resources), designated for cytokine mRNA and protein analysis, were then immediately snap-frozen and stored at −80°C for subsequent assays.

### Quantitative real-time PCR (qPCR)

Total RNA was extracted from distal colon tissues using TRIzol reagent. cDNA was synthesized using a reverse transcription kit. qPCR was performed to determine the mRNA expression levels of IL-10, IL-17, and IL-22. Target gene expression was normalized to the housekeeping gene beta-actin using the 2^(-ΔΔct) method.

### Western blot analysis

Total protein was extracted from distal colon tissues and quantified using a BCA protein assay kit. Equal amounts of protein (30–50 μg) were separated by SDS-PAGE and transferred onto PVDF membranes. After blocking with 5% non-fat milk, membranes were incubated overnight at 4°C with primary antibodies against TLR4 and MyD88. After washing, membranes were incubated with HRP-conjugated secondary antibodies. Protein bands were visualized using enhanced chemiluminescence (ECL) and quantified via densitometry using ImageJ software.

### Statistical analysis

Data are presented as mean ± standard deviation (SD). For the fold-change data derived from qPCR and Western blot analyses, a log_2_ transformation was applied prior to statistical testing to satisfy assumptions of normality. Statistical comparisons between multiple groups were performed using one-way ANOVA followed by Tukey’s post-hoc test. The Shapiro-Wilk test was utilized to evaluate the normality of the data, and Levene's test was applied to assess the homogeneity of variance. *P*-values < 0.05 were considered statistically significant. Statistical analyses were performed using GraphPad Prism (v9.0) and R software.

## Results

### Adult-sourced FMT partially ameliorates microbial alpha diversity in juvenile mice

To evaluate the impact of ABX and subsequent FMT on microbial alpha diversity, we evaluated the Chao1 and Shannon indices (Fig 1a). ABX administration (EXP group) precipitated a profound contraction in species richness and community evenness compared to the CON group (Chao1 & Shannon, *P* < 0.001). Conversely, FMT intervention facilitated a substantial structural rescue of the microbiome. While the Chao1 index indicated a significant but partial restoration of microbial richness following FMT (*P* = 0.015 vs. EXP), the Shannon index demonstrated a robust recovery of overall community diversity (*P* = 0.0012 vs. EXP), reaching levels comparable to the baseline CON group (*P* = 0.16). These findings suggest that FMT increased community diversity in juvenile recipients (n = 6 per group).

**Fig 1:**
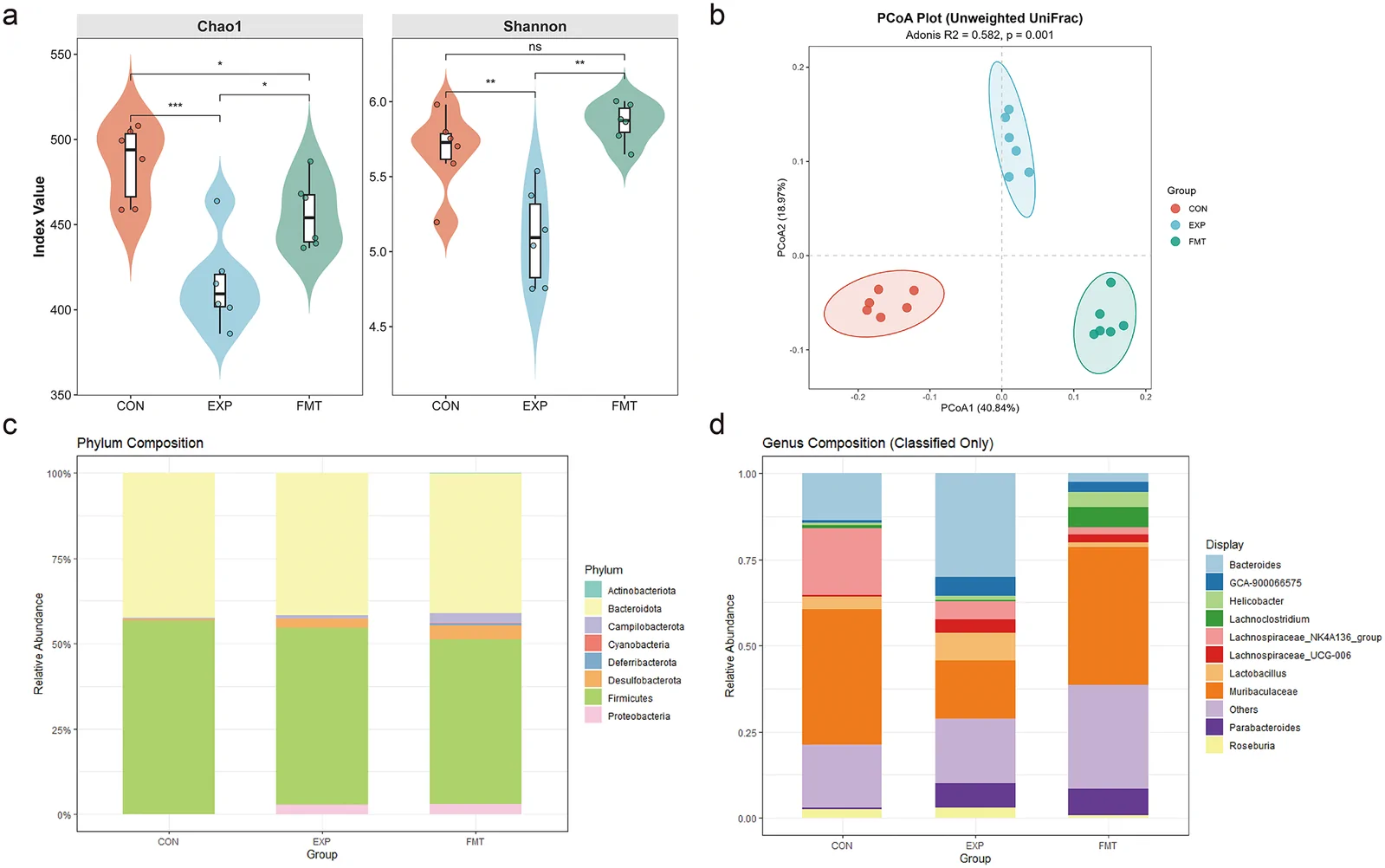
Adult-sourced FMT modulates microbial alpha and beta diversity. (a) Alpha diversity measured by Chao1 richness and Shannon diversity indices across CON, EXP, and FMT groups (*P < 0.05, **P < 0.01, ***P < 0.001, ns = not significant; n = 6 per group). (b) Principal Coordinate Analysis (PCoA) plot based on Unweighted UniFrac distance (Adonis R^2^ = 0.582, *P* = 0.001). (c) Community composition at the Phylum level. (d) Community composition at the Genus level (classified only).

### FMT reshapes the antibiotic-disrupted gut microbiota into a novel and mature ecological state

To evaluate the structural divergence of the gut microbiota across the experimental cohorts, we performed Principal Coordinate Analysis (PCoA) based on both unweighted and weighted UniFrac distance metrics. The unweighted UniFrac PCoA analysis (Fig 1b) revealed distinct, non-overlapping clustering of the microbial communities among the CON, EXP, and FMT groups, which was statistically validated by PERMANOVA testing (Adonis R^2^ = 0.582, *P* = 0.001). This robust structural segregation was further corroborated by the weighted UniFrac distance metric (S1 File), which accounts for the relative abundance of dominant taxa (Adonis R^2^ = 0.618, *P* = 0.001). Collectively, these beta diversity analyses demonstrate that while antibiotic exposure profoundly disrupted the baseline microbiota, the subsequent FMT intervention did not merely revert the microbiome to its original configuration, but rather effectively reshaped the microbial landscape into a distinctly new community state.

Taxonomic composition analysis at the phylum and genus levels (Fig 1c, 1d) elucidated the profound microbial remodeling induced by ABX exposure and subsequent FMT. At the phylum level, while Firmicutes and Bacteroidota remained dominantly conserved across all cohorts, EXP group precipitated community dysbiosis, characterized by a slight reduction in Firmicutes and a disproportionate expansion of Proteobacteria. Following FMT, Desulfobacterota and Campilobacterota were detected at higher relative abundance than in either CON or EXP. Because the donor inoculum was not sequenced, we cannot confirm that these taxa originated from the donor; their appearance after transplantation is consistent with, but does not demonstrate, a donor origin. At a higher phylogenetic resolution, the genus-level analysis revealed that ABX drove a massive expansion of Bacteroides alongside a drastic depletion of normative core taxa such as the *Lachnospiraceae_NK4A136_group* and *Muribaculaceae*. Remarkably, FMT intervention not only constrained the ABX-induced *Bacteroides* overgrowth and promoted a robust resurgence of *Muribaculaceae* beyond CON levels, but it also established an entirely novel structural paradigm. This was evidenced by the appearance of genera that were rare or undetected in CON and EXP (for example *Helicobacter* and *Lachnoclostridium*), which have been reported as characteristic of the mature murine microbiota [22–25] and a notable enrichment of diverse, low-abundance taxa (the “Others” fraction), collectively driving the juvenile gut microbiome toward a more complex and mature ecological state.

To further understand microbiota resilience and its dynamics following antibiotic treatment and FMT, we identified the number of unique and shared OTUs among groups. The OTU overlap analysis revealed that while ABX drastically reduced shared baseline OTUs, subsequent FMT facilitated a partial overlap of OTUs between the CON and FMT groups, indicating that FMT supported the re-establishment of certain baseline microbiota members while simultaneously introducing a distinct suite of unique OTUs not present in the original juvenile gut (S1 File).

### Identification of taxonomic biomarkers associated with the community shifts

To delineate the specific taxa associated with the structural divergence across groups, we performed LEfSe coupled with a cladogram analysis (Fig 2a, 2b). In the undisturbed juvenile gut (CON group), the microbiome was significantly enriched with normative keystone commensals, predominantly *Muribaculaceae* and *Lachnospiraceae_NK4A136_group* (Fig 2a). Following antibiotic exposure, the EXP group exhibited a drastic ecological shift, characterized by a massive, statistically significant overgrowth of the genus *Bacteroides* and lineages within the class Bacilli (specifically *Lactobacillus*) (Fig 2a, 2c). Notably, the relative abundance of *Muribaculaceae* and *Lachnospiraceae_NK4A136_group* was severely depleted in the EXP cohort (Fig 2c).

**Fig 2:**
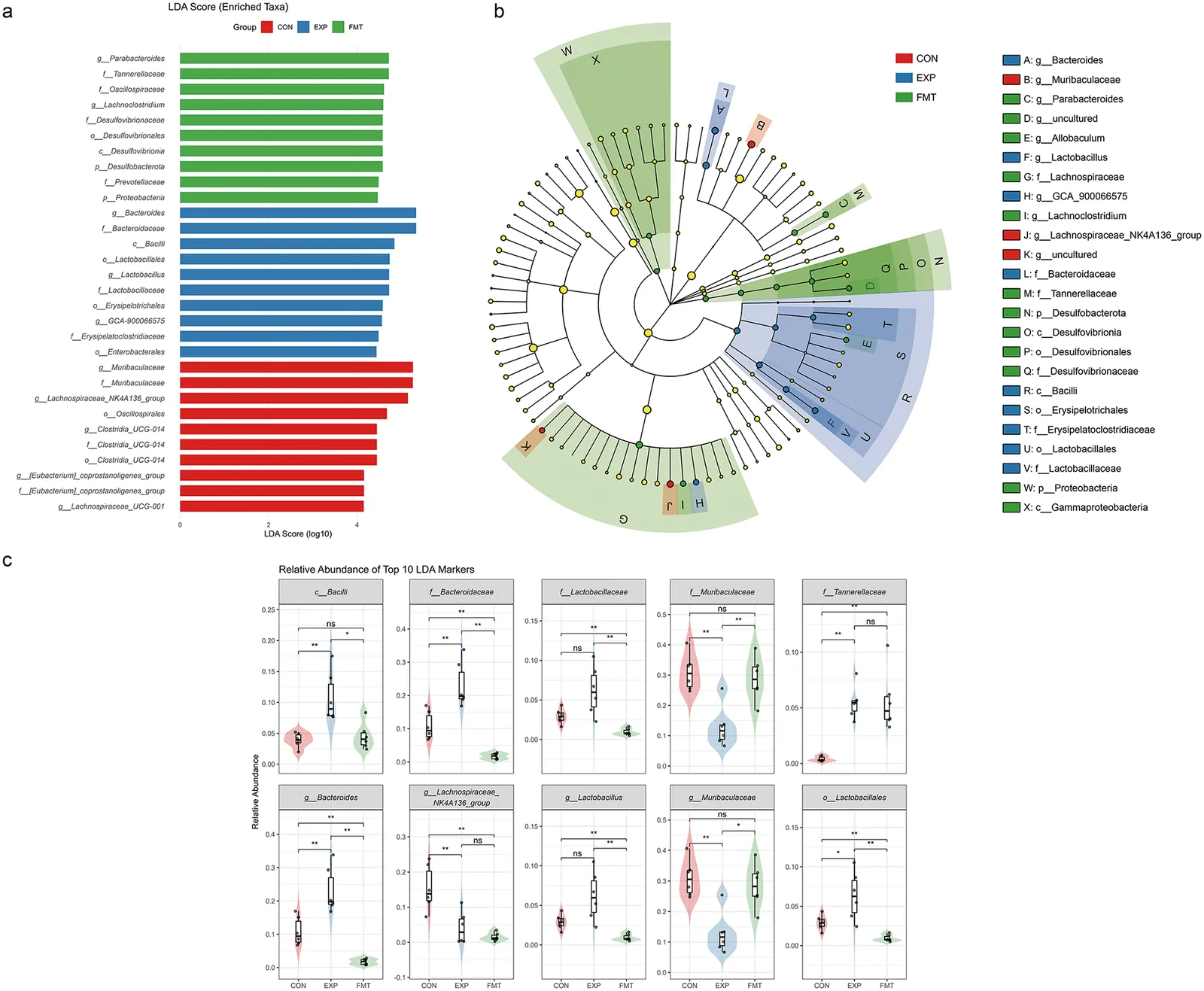
LEfSe analysis identifies taxonomic biomarkers of microbial modulation. (a) Histogram of Linear Discriminant Analysis (LDA) scores for differentially abundant bacteria (LDA > 3.0, *P* < 0.05). (b) Cladogram showing the evolutionary relationships of the identified biomarkers. (c) Relative abundance comparison of the Top 10 LDA markers among the three groups (*P < 0.05, **P < 0.01, ns = not significant; n = 6 per group).

Subsequent FMT intervention was associated with a marked change in community structure relative to EXP. FMT successfully suppressed the antibiotic-induced expansion of *Bacteroides* and Lactobacillales, reducing their relative abundances to levels equal to or lower than the baseline CON group (Fig 2c). Concurrently, FMT facilitated the complete recovery of *Muribaculaceae*, returning its abundance to levels statistically indistinguishable from the healthy CON mice (Fig 2c). Crucially, the LEfSe and phylogenetic analyses revealed that the FMT group developed an entirely distinct biomarker profile, heavily enriched with taxa frequently associated with a mature intestinal microbiome, including *Parabacteroides*, *Lachnoclostridium*, *Tannerellaceae*, and sulfur-reducing bacteria belonging to the phylum Desulfobacterota (Fig 2a, 2b).

### FMT partially attenuates the antibiotic-induced increase in colonic IL-17 and IL-22 transcripts

To evaluate the immunological consequences of antibiotic-induced dysbiosis and subsequent FMT intervention, we quantified the mRNA expression of key cytokines in the distal colon. As shown in Fig 3a, early-life ABX exposure produced a significant increase relative to CON in colonic transcript levels of IL-10, IL-17, and IL-22 (*P* < 0.01, *P* < 0.0001, and *P* < 0.001, respectively). Of these, IL-17 and IL-22 are effector cytokines of the mucosal type-17/type-22 response, whereas IL-10 is immunoregulatory. Following the administration of adult-sourced FMT, this mucosal immune hyperactivation was substantially mitigated. Specifically, FMT significantly reduced the expression levels of the effector cytokines IL-17 and IL-22 compared to the EXP cohort (*P* = 0.036, and *P* = 0.024, respectively). Interestingly, while IL-10 expression remained elevated in the FMT group relative to controls (*P* = 0.014), it was not significantly different from the EXP group (*P* = 0.068), suggesting a sustained regulatory immune tone. These data indicate that FMT partially attenuates the antibiotic-associated increase in IL-17 and IL-22 transcripts exacerbated by antibiotic stress.

**Fig 3:**
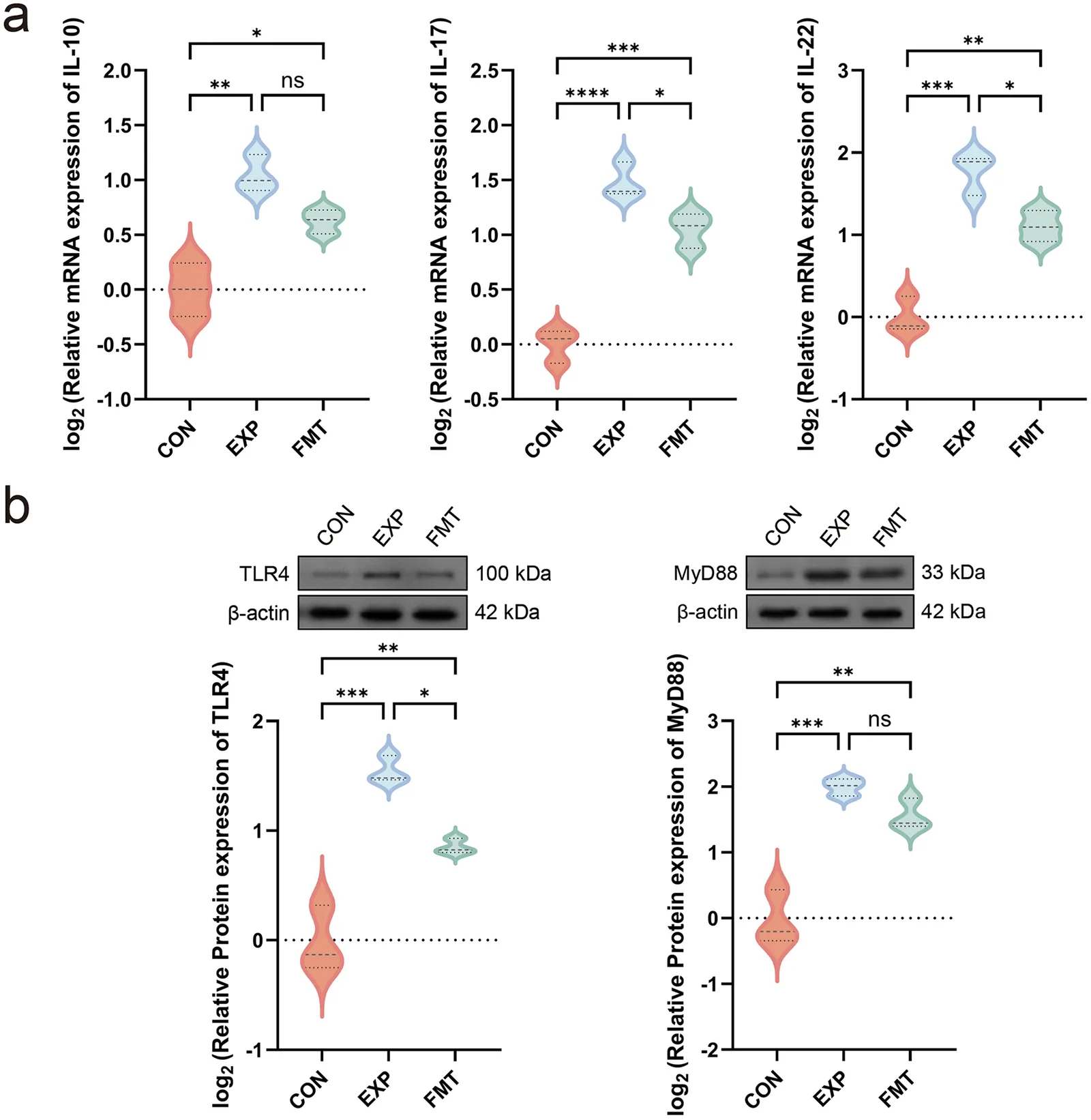
Colonic cytokine transcript levels and TLR4/MyD88 protein levels in CON, EXP and FMT mice. (a) Relative mRNA expression levels of IL-10, IL-17, and IL-22 in distal colon tissue determined by qPCR. (b) Representative Western Blot bands for TLR4, MyD88, and beta-actin. (c) Densitometric quantification of TLR4 and MyD88 protein expression, normalized to beta-actin (*P < 0.05, ** *P* < 0.01, *** *P* < 0.001, **** *P* < 0.0001, ns = not significant; data are presented as mean ± SD, n = 3 per group).

### TLR4 and MyD88 protein levels after FMT

Given the critical role of microbial sensing in mucosal immunity, we investigated the TLR4/MyD88 signaling axis to elucidate the molecular mechanisms underlying the immune-modulatory effects of FMT. Western blot analysis (Fig 3b) demonstrated that early-life ABX exposure induced a severe hyperactivation of this pathway, characterized by a substantial increase in the protein expression of both the pattern recognition receptor TLR4 and its downstream adaptor MyD88 in the colonic tissue (*P* < 0.001 vs. CON group). Notably, FMT intervention successfully attenuated this signaling cascade, driving a significant downregulation of TLR4 protein expression (*P* = 0.011 vs. EXP group). Although MyD88 levels exhibited a downward trend following FMT, the reduction did not reach statistical significance compared to the EXP group. Collectively, these findings are consistent with an association between FMT and lower TLR4 protein levels.

## Discussion

The neonatal and juvenile periods are critical for the assembly of the gut microbiota and the concomitant maturation of the host immune system. Our study demonstrates that early-life antibiotic exposure precipitates a profound ecological alteration, triggering a state of “immune stress” characterized by the overactivation of the TLR4/MyD88 signaling axis and a surge in effector cytokines. Notably, we found that adult-sourced FMT effectively ameliorates this developmental trajectory, providing a microbial community whose establishment was accompanied by a partial reduction in colonic TLR4 and MyD88 protein and in IL-17 and IL-22 transcript levels. Whether this represents a return toward mucosal homeostasis was not assessed, as barrier function and tissue architecture were not evaluated.

A critical finding in our study is that FMT did not merely reconstitute the pre-antibiotic juvenile state; rather, it generated a new, structurally distinct, and mature microbial community. In normal conditions, the juvenile gut is dominated by highly protective core commensals such as *Muribaculaceae* and *Lachnospiraceae*. Following ABX administration, these protective taxa experienced a drastic relative reduction, and the vacated ecological niches were aggressively monopolized by *Bacteroides*. While FMT successfully suppressed this *Bacteroides* overgrowth and rescued specific keystone families like *Muribaculaceae*, it did not restore all original juvenile taxa, such as the *Lachnospiraceae_NK4A136_group*. Instead, the “cleared” gut environment provided an optimal engraftment window for adult-sourced donor microbiota. The robust colonization of mature signature taxa—such as *Lachnoclostridium*, *Parabacteroides*, *Tannerellaceae*, and *Desulfobacterota*—indicates a significant upgrade in ecological complexity. Although direct sequencing of the adult donor feces was not performed, these specific taxonomic signatures are widely documented in the literature as hallmark indicators of a mature murine intestinal microbiome [22–25]. This transition is characterized by a shift toward a highly complex, strictly anaerobic consortium equipped for advanced complex carbohydrate degradation, an ecological niche predominantly filled by Tannerellaceae and *Parabacteroides* [26]. Therefore, the severe ecological vacuum created by antibiotic exposure provides a permissive window, allowing the adult-sourced FMT to rapidly establish a structurally distinct and mature microecosystem.

Immunologically, ABX treatment induced a massive upregulation of the TLR4/MyD88 signaling pathway at the protein level, accompanied by a surge in IL-17 and IL-22 mRNA expression (Fig 3a). This immune stress is highly likely driven by the dysbiotic expansion of Gram-negative opportunistic pathogens that shed elevated levels of lipopolysaccharide (LPS). The binding of LPS to TLR4 recruits the adaptor protein MyD88, initiating an intracellular cascade that culminates in the transcription of effector cytokines [27]. Remarkably, FMT mitigated this stress by significantly downregulating TLR4 expression and suppressing IL-17 and IL-22. Interestingly, the mRNA expression of IL-10—a potent anti-inflammatory cytokine—remained elevated following FMT. Alternatively, the elevation of IL-10 in the EXP group may represent a counter-regulatory response to the perturbed environment, which partially resolves and is numerically lower after FMT as that environment partially recovers. This would also explain the low basal levels of IL-10 in the CON group. It is important to note, however, that while FMT significantly attenuated the expression of these inflammatory markers, it achieved a partial amelioration rather than a complete restoration to baseline Control levels. All five endpoints show FMT means between EXP and CON, which supports the explanation that the relatively short 4-day intervention window achieved only a partial recovery. Future studies should employ a longer FMT course with several sampling time points to assess the long-term stability of the transplanted community, alongside the barrier and LPS/SCFA assays already listed.

However, the taxonomic profile of the FMT group presents a fascinating mechanistic paradox. The FMT recipients exhibited an enrichment of specific Gram-negative taxa, notably Proteobacteria, Desulfobacterota (including Desulfovibrionaceae), and Tannerellaceae. Since these taxa inherently possess LPS, their colonization would canonically be expected to upregulate TLR4 signaling. The observed down-regulation of TLR4 alongside the enrichment of these LPS-bearing bacteria suggests an ambiguous, potentially symbiotic ecological shift rather than a straightforward beneficial restoration. This suggests a complex mechanistic paradox where structural variations in mature LPS may act as TLR4 antagonists, or alternative tolerogenic pathways override endotoxin signaling. This paradox may be explained by structural variations in the LPS produced by different mature taxa, which could exhibit varying immunogenicities and act as TLR4 antagonists rather than agonists. Alternatively, the expansion of sulfur-reducing bacteria like Desulfobacterota closely parallels the maturation of the intestinal mucus layer [28], and competing regulatory mechanisms, such as SCFA production by other engrafted species, might override the LPS signal. Furthermore, we noted a subtle expansion of *Helicobacter* in the FMT group (Fig 1d). While often commensal in mice, its appearance highlights the double-edged nature of undefined FMT consortia and the potential risk of transferring pathobionts.

Several limitations of this study warrant consideration. First, the health status of the mice was monitored qualitatively, and systematic quantitative tracking of body weight and food intake was not recorded. Second, the lack of 16S sequencing data for the donor inoculum restricts our ability to directly confirm the exact origin and engraftment rate of adult donor strains. Additionally, a single pooled inoculum from five donors was given to all recipients, meaning the donor community represents a single experimental unit. Third, the study lacks an age-matched juvenile donor comparator, limiting definitive claims about the unique advantages of an adult-sourced “mature template.” Finally, the reliance on a single reference gene (beta-actin) for qPCR normalization is suboptimal, and the selection of n = 3 mice for molecular endpoints limits the detection of smaller differences in immune responses. Future studies must incorporate functional barrier assays and quantify luminal LPS or SCFAs to resolve the mechanistic paradox of TLR4 down-regulation alongside the enrichment of specific Gram-negative taxa.

## Conclusion

This study demonstrates that adult-sourced FMT effectively mitigates early-life antibiotic-induced intestinal immune stress associated with a partial reversal of the antibiotic-induced reduction in alpha diversity and with a shift toward a distinct state. Rather than merely reverting the microbiome to its naive juvenile baseline, the FMT intervention successfully suppresses the opportunistic overgrowth of Bacteroides and facilitates the engraftment of mature-signature taxa. Concurrently, this microbial remodeling attenuates the antibiotic-induced hyperactivation of the mucosal TLR4 signaling cascade, thereby suppressing the expression of pro-inflammatory effector cytokines (IL-17 and IL-22) while sustaining immunoregulatory IL-10 production. These findings provide a basis for further investigation of donor-age-related effects on microbial and mucosal immune recovery after early-life antibiotic exposure.

## Supporting information

S1 File 16S rRNA sequencing supplementary analysis results. This single PDF document contains supplementary figures evaluating microbial alpha and beta diversity, as well as OTU overlap. Alpha diversity materials include Observed OTUs rarefaction curves, Rank-Abundance curves, Group Rank-Abundance, and Group OTU Venn diagrams. Beta diversity materials include Group NMDS plots, PCA group plots, Weighted UniFrac PCoA plots, and UPGMA clustering trees.
(PDF)

S2 File Quantitative real-time PCR (qPCR) and Western blot data. This Excel workbook includes the primer information, melting curves, raw Ct values, and calculation spreadsheets for the mRNA expression levels of IL-10, IL-17, and IL-22, along with the densitometric data for the Western blots of β-actin, TLR4, and MyD88.
(XLSX)

S3 File Uncropped Western blot images. This single PDF document contains the original, uncropped, and unadjusted chemiluminescence images for TLR4, MyD88, and β-actin utilized for densitometric quantification.
(PDF)
