# Supplementary material for: Adult-sourced fecal microbiota transplantation ameliorates early-life antibiotic-induced intestinal immune stress in juvenile mice associated with the modulation of the TLR4/MyD88 signaling pathway
Source: PLoS One. 2026 Oct 13;21(10):e0359634. doi: 10.1371/journal.pone.0359634 (PMC13660878; doi:10.1371/journal.pone.0359634)

Supplementary Figure 1: Observed OTUs rarefaction curves.

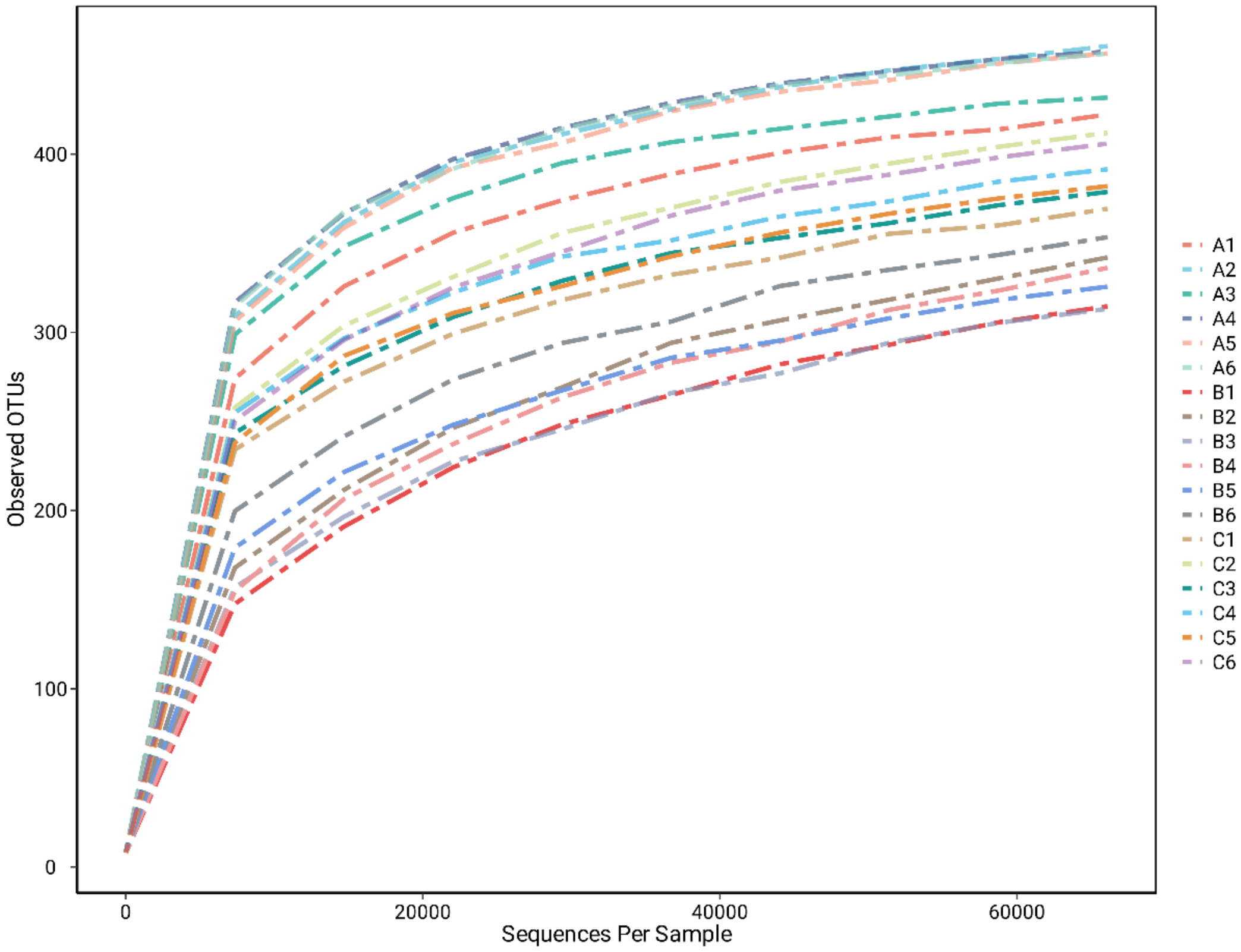

Supplementary Figure 2: Rank-abundance curves of all samples.

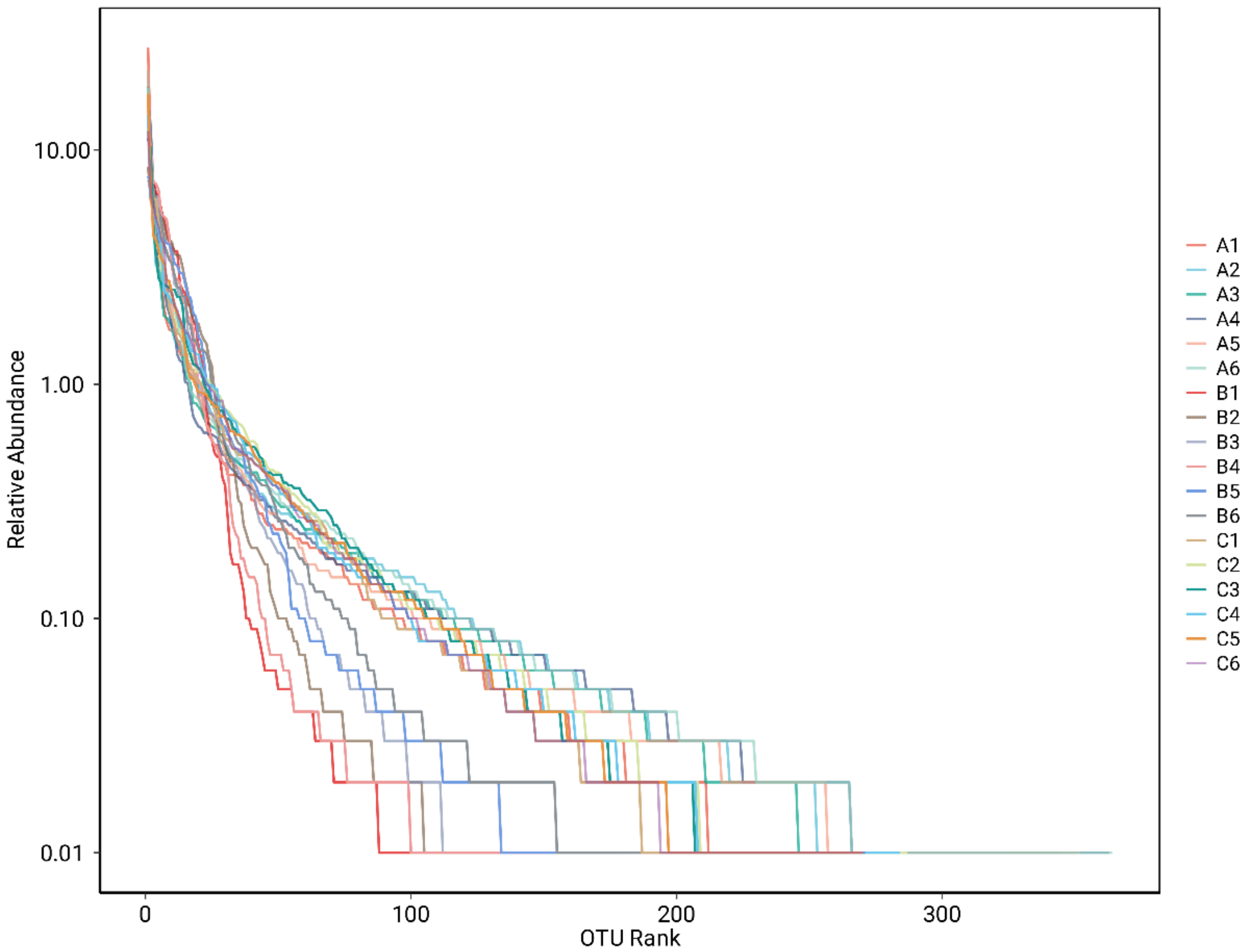

Supplementary Figure 3: Group-level rank-abundance curves.

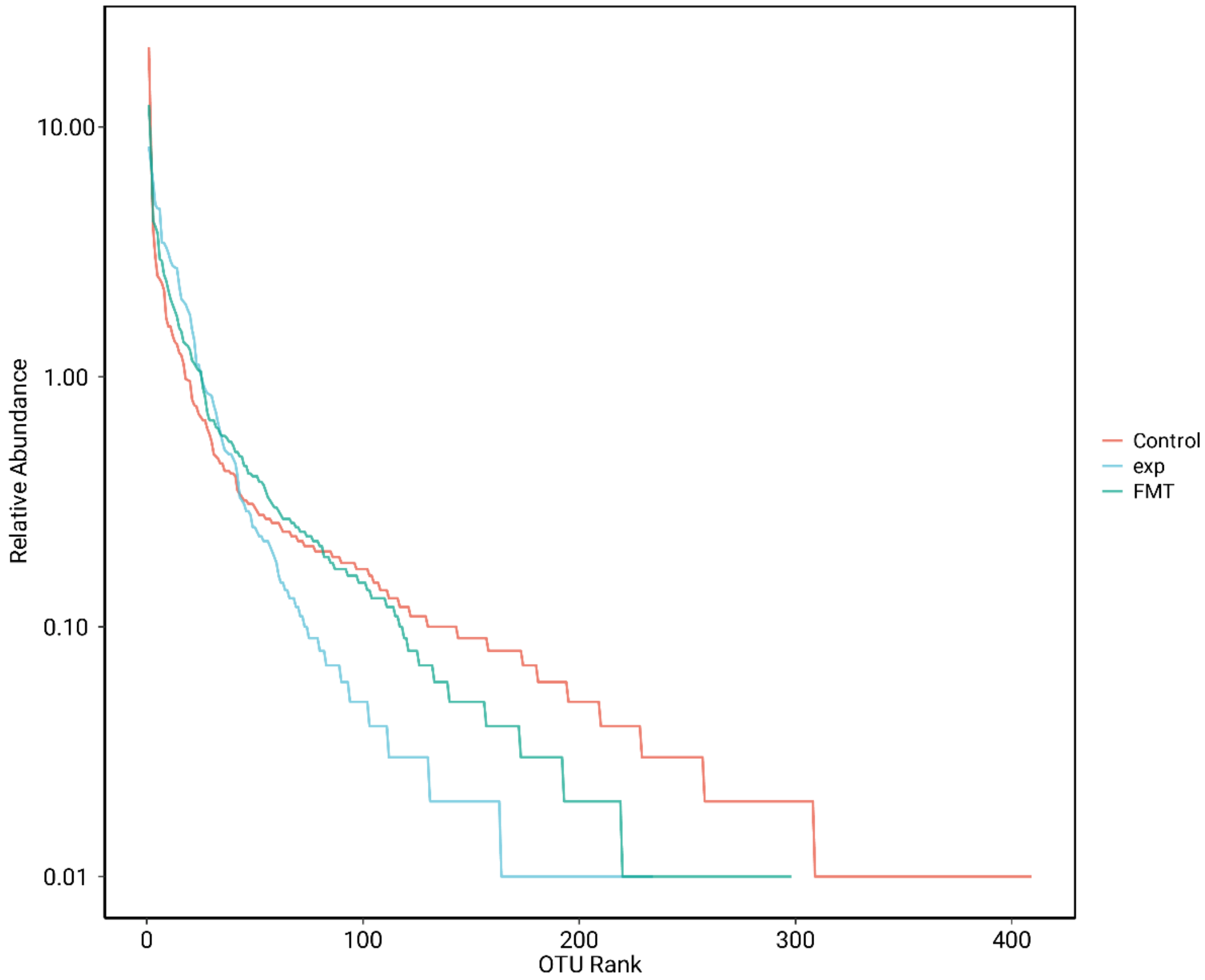

Supplementary Figure 4: Group OTU Venn diagram.

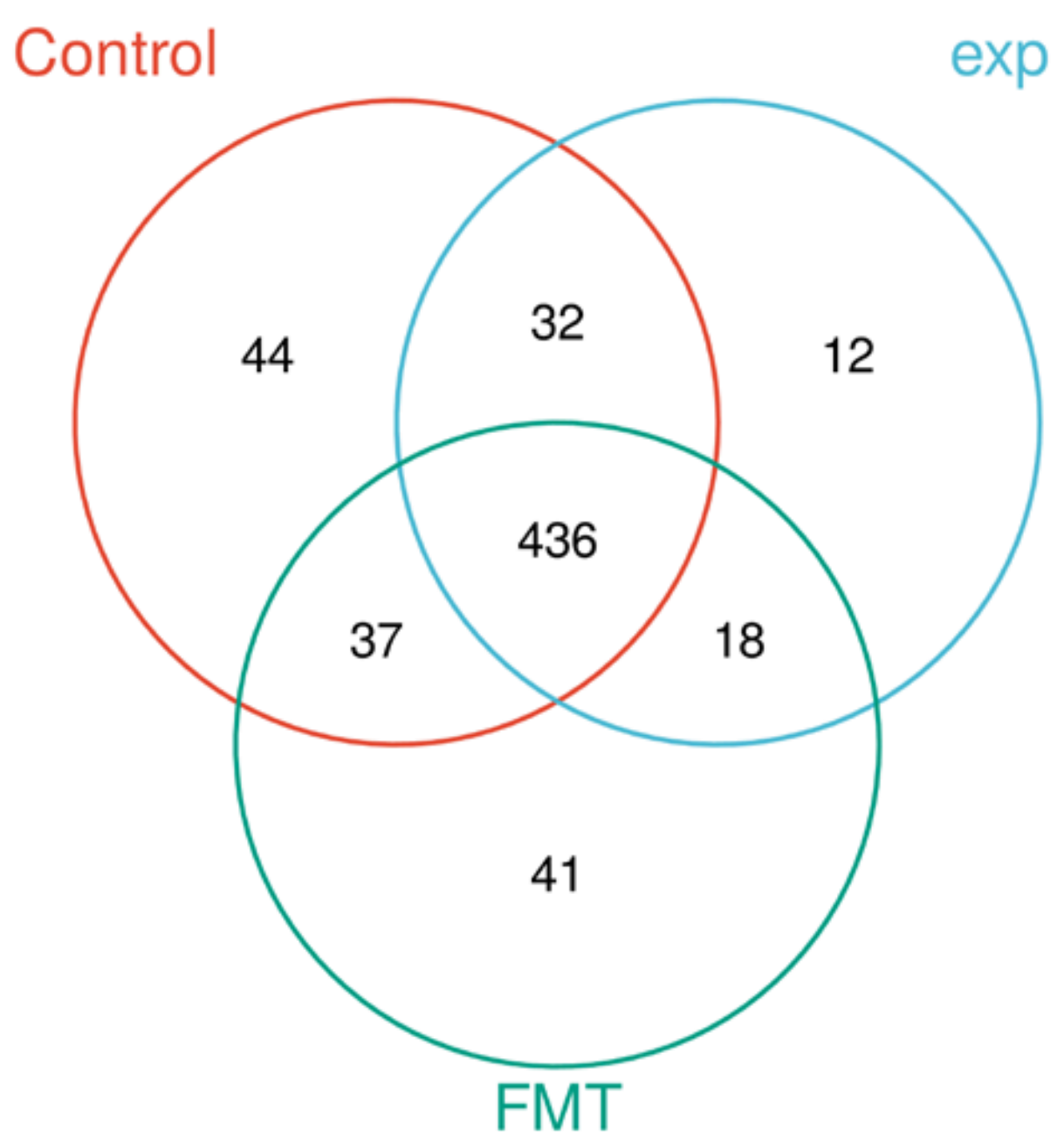

Supplementary Figure 5: Principal Component Analysis (PCA) plot.

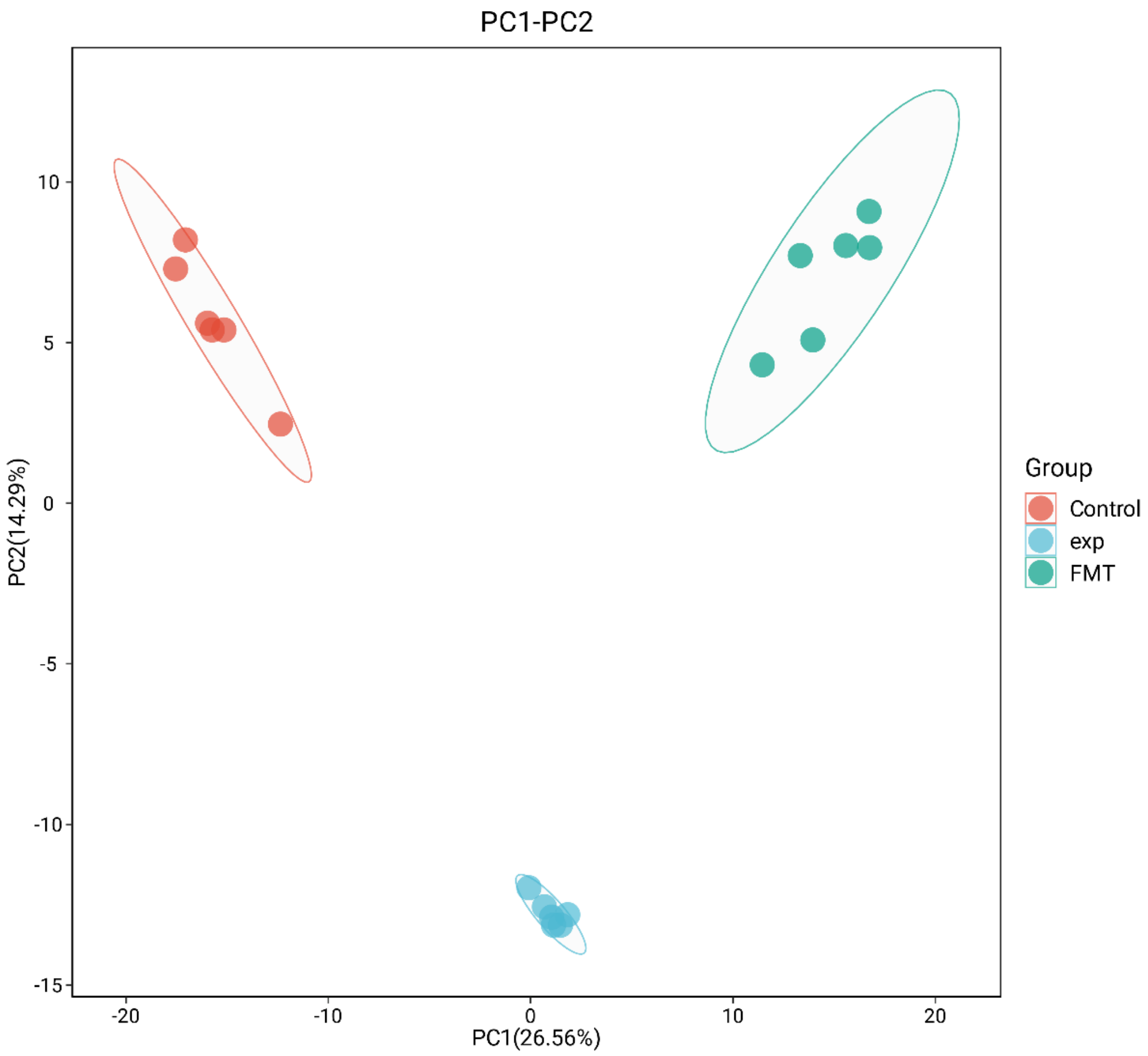

Supplementary Figure 6: Non-metric Multidimensional Scaling (NMDS) plot.

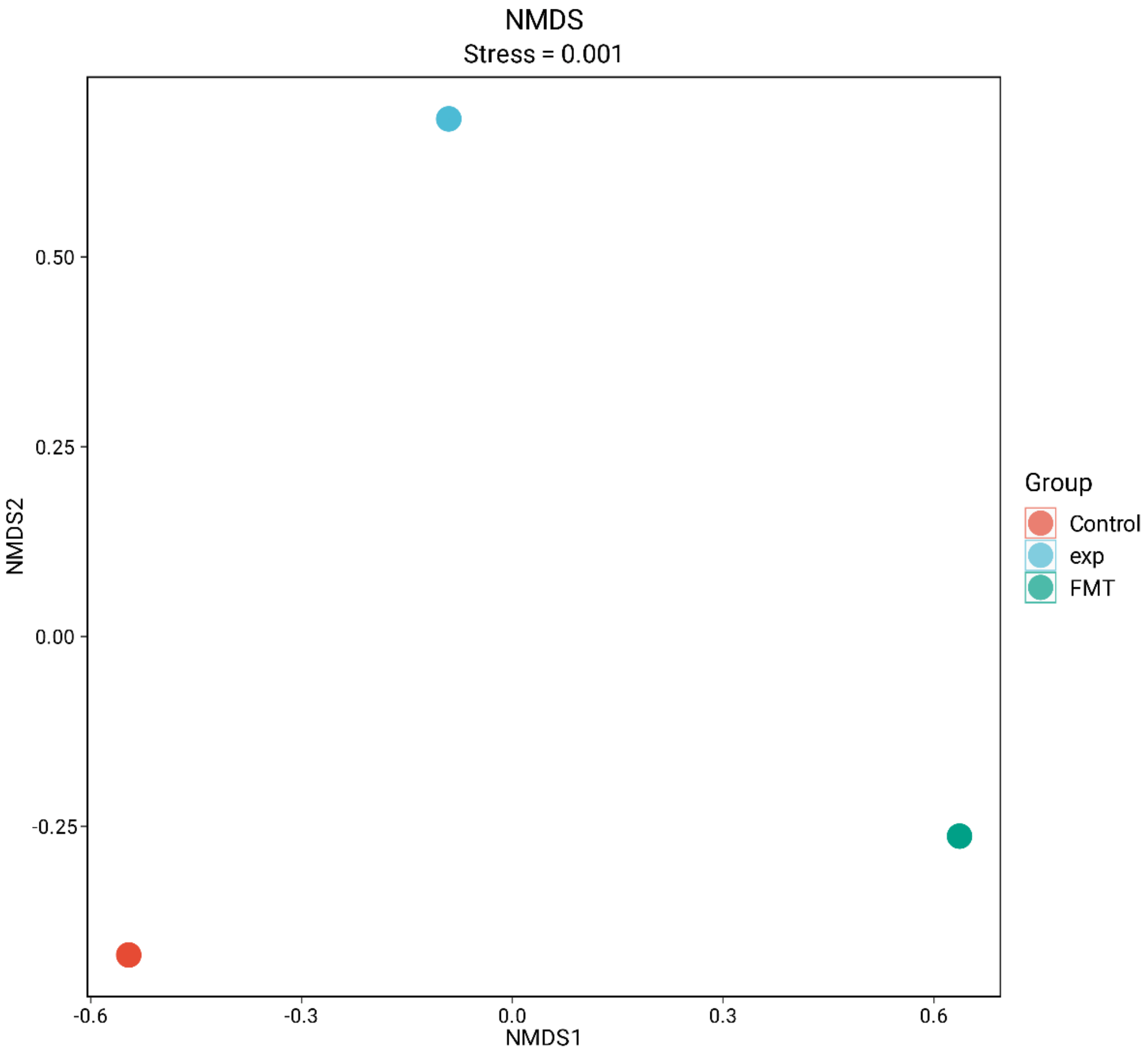

Supplementary Figure 7: Principal Coordinate Analysis (PCoA) based on weighted UniFrac distances.

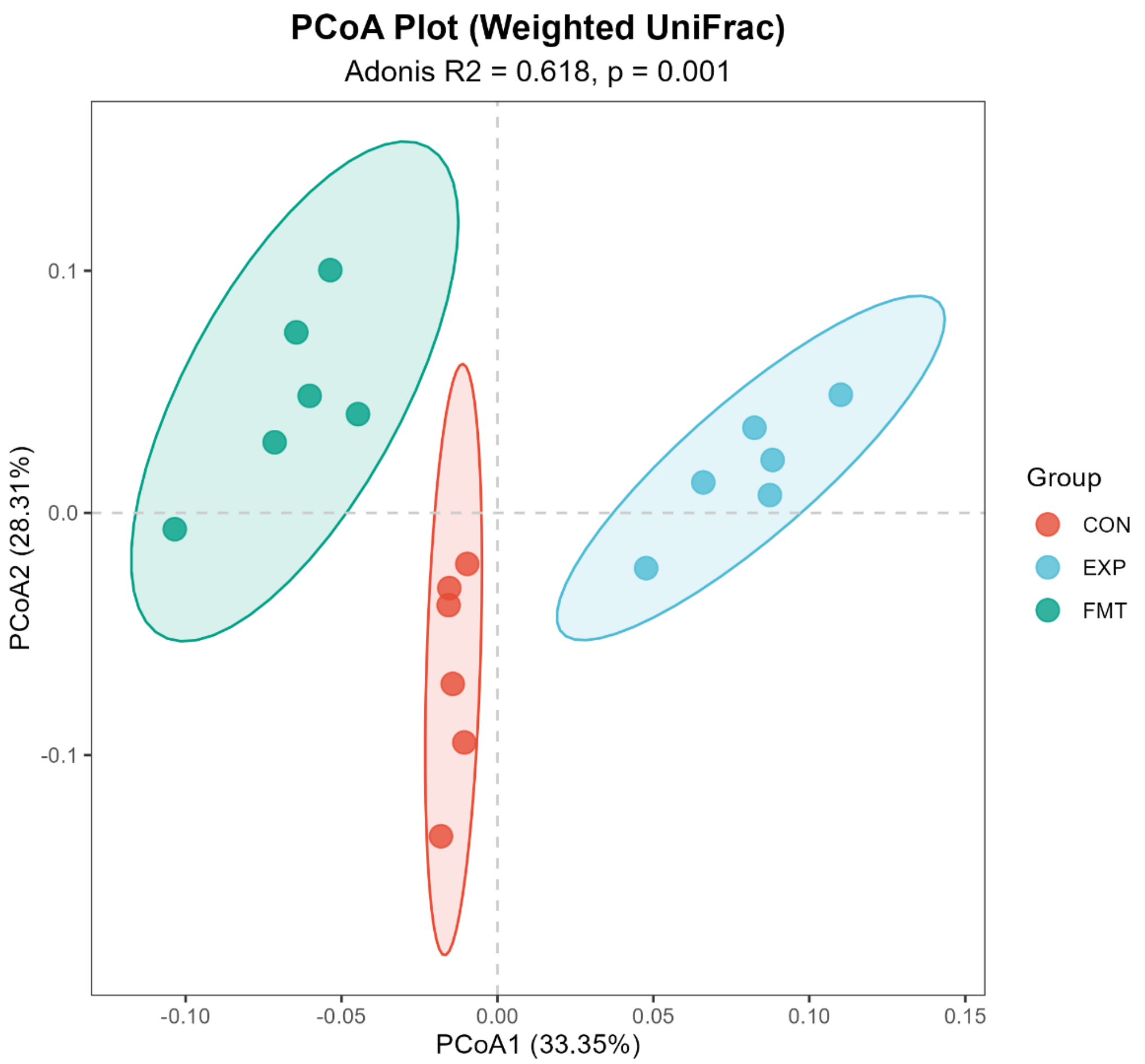

Supplementary Figure 8: UPGMA clustering tree.

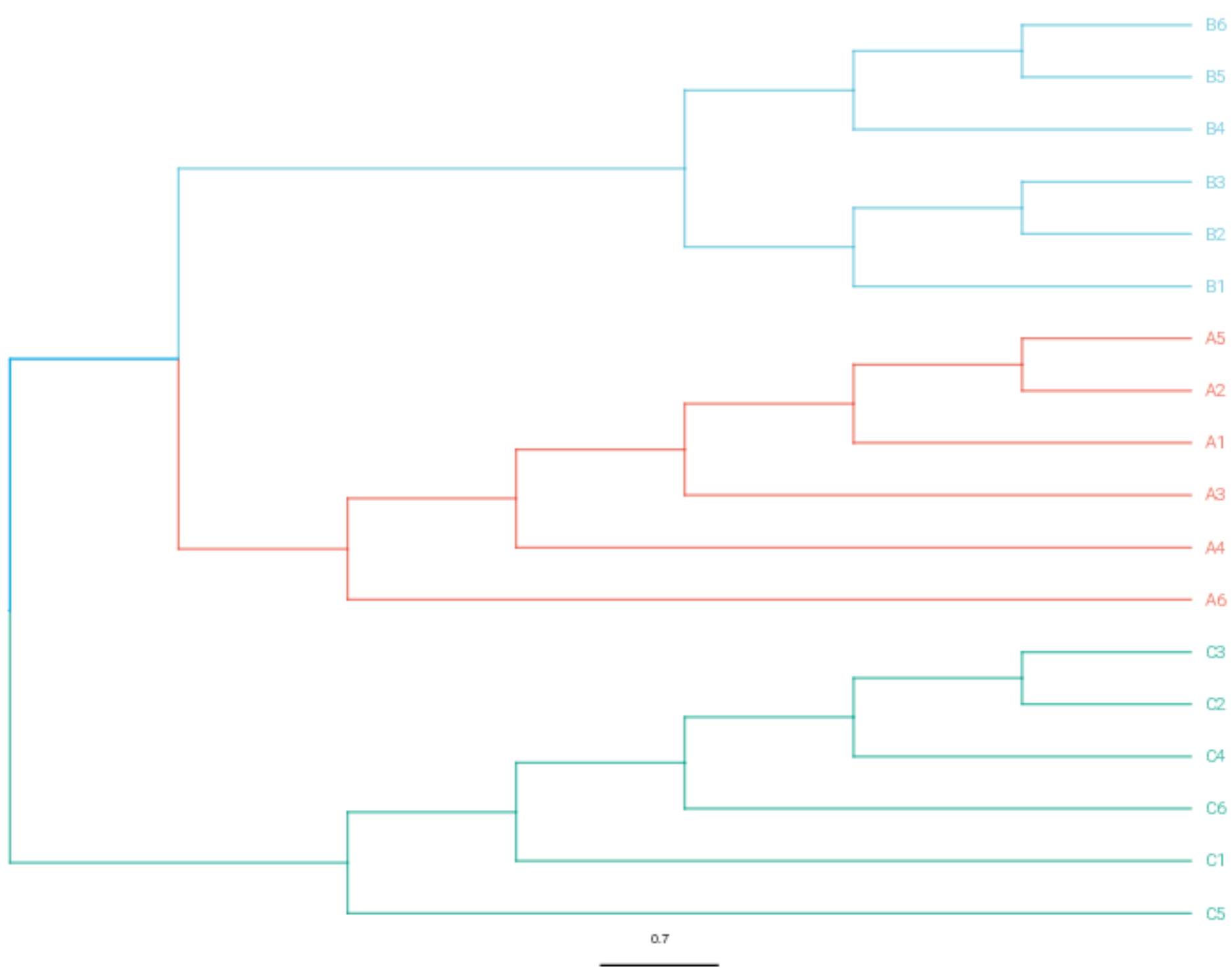

Supplement: Supplementary file 1 [file pone.0359634.s001.pdf]
