# Supplementary material for: Adult-sourced fecal microbiota transplantation ameliorates early-life antibiotic-induced intestinal immune stress in juvenile mice associated with the modulation of the TLR4/MyD88 signaling pathway
Source: PLoS One. 2026 Oct 13;21(10):e0359634. doi: 10.1371/journal.pone.0359634 (PMC13660878; doi:10.1371/journal.pone.0359634)
Supplement: Supplementary file 2 [file pone.0359634.s003.pdf]

Figure3b

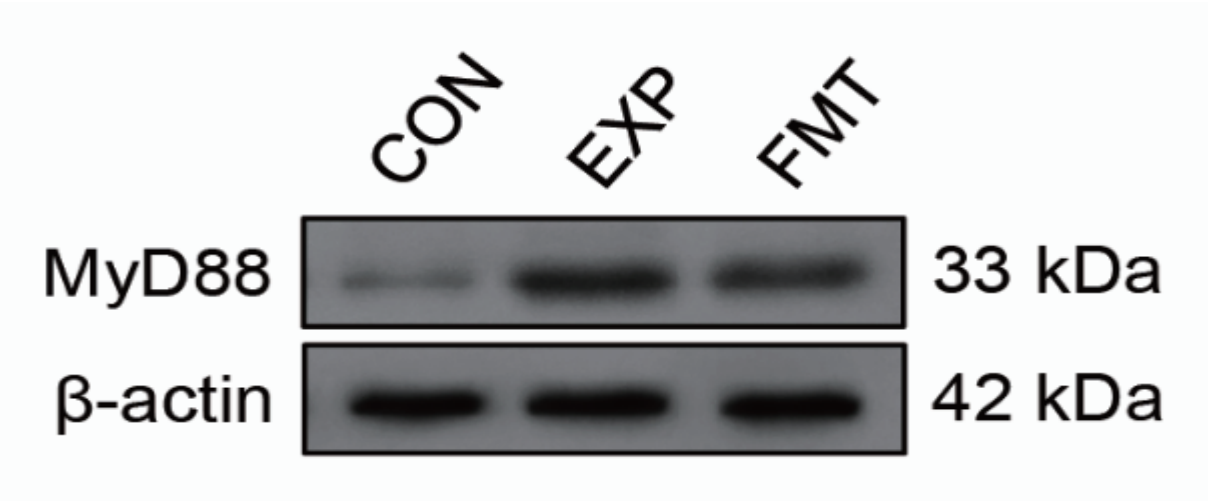
CON
EXP
FMT
MyD88
33 kDa
β-actin
42 kDa

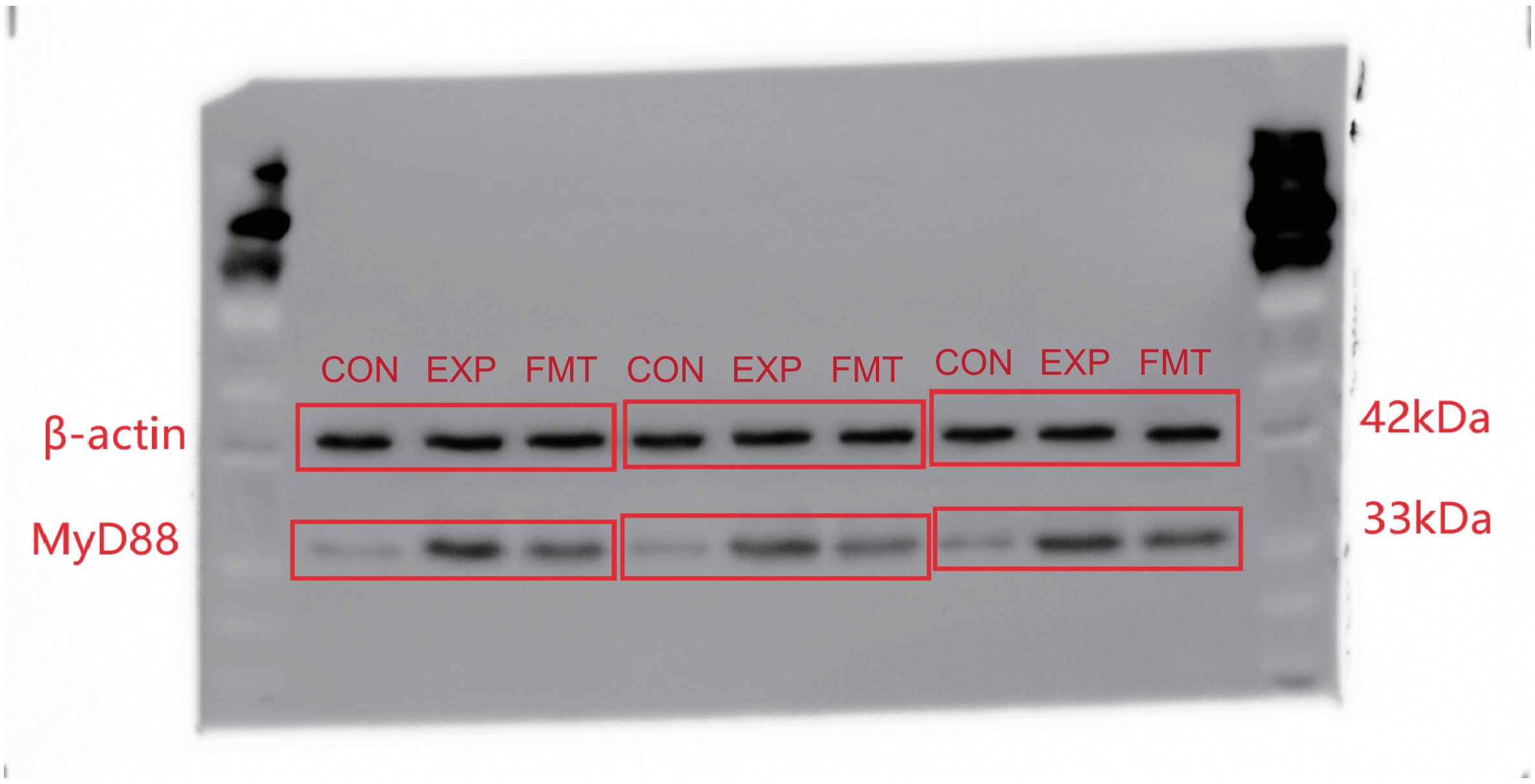
CON EXP FMT CON EXP FMT CON EXP FMT
β-actin
MyD88
42kDa
33kDa

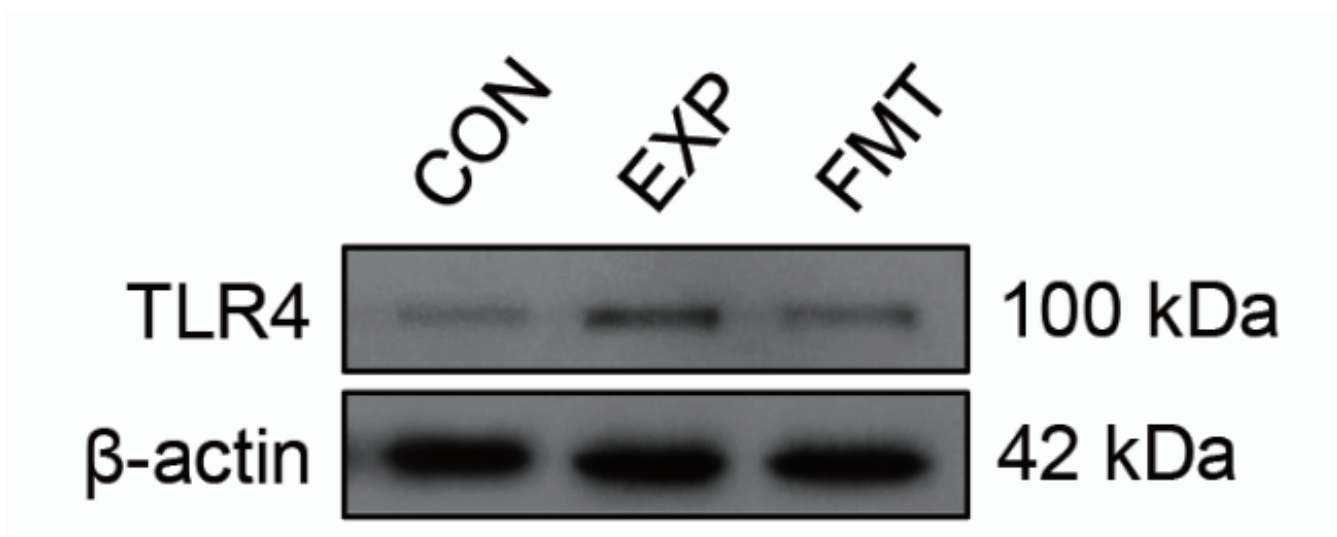
CON
EXP
FMT
TLR4
100 kDa
β-actin
42 kDa

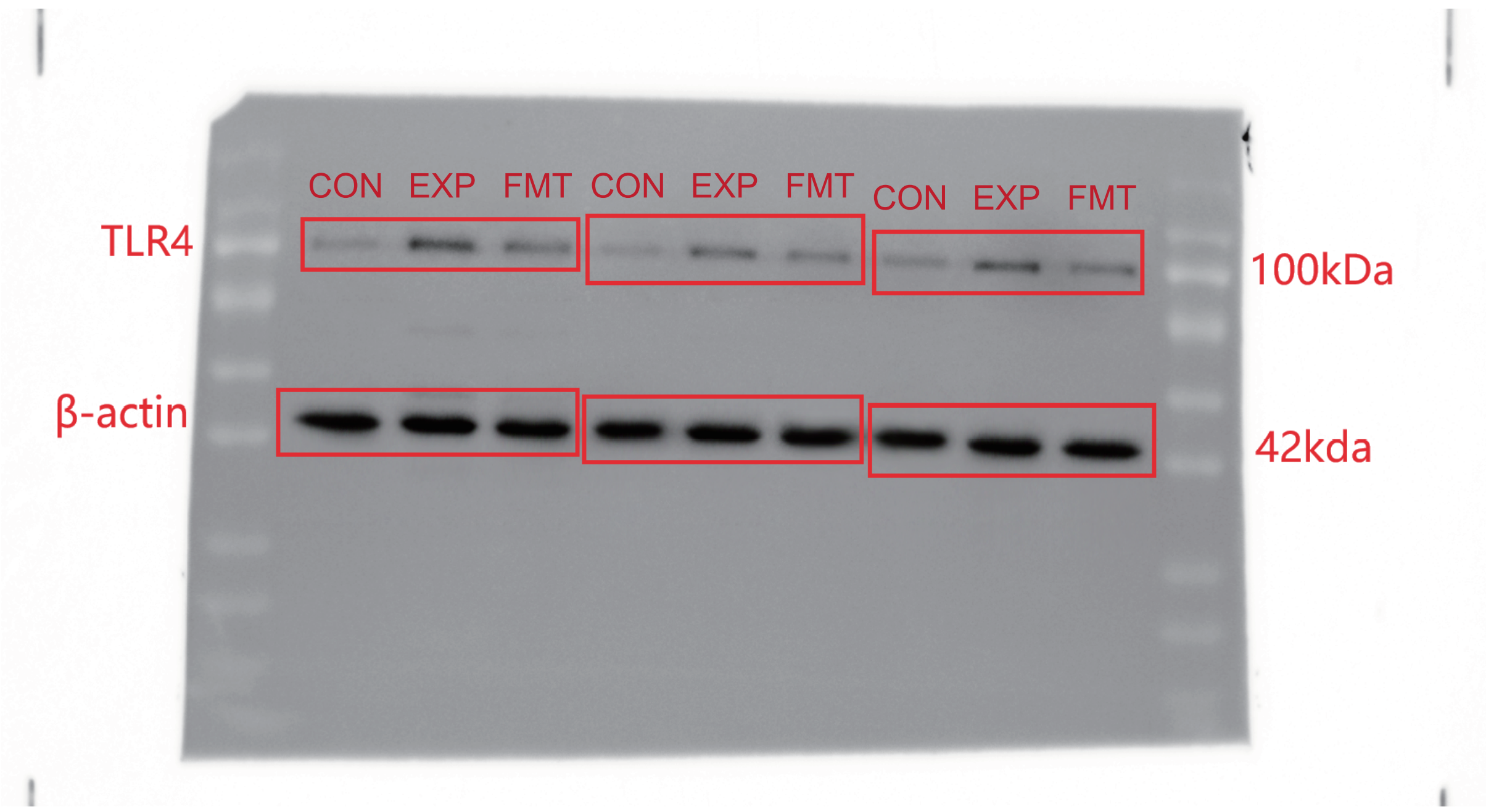
CON EXP FMT CON EXP FMT CON EXP FMT
TLR4
β-actin
100kDa
42kda
